# Application of Bayesian modeling for diagnostic assays of *Mycobacterium avium* subsp. *paratuberculosis* in sheep and goats flocks

**DOI:** 10.1186/s12917-022-03141-7

**Published:** 2022-01-18

**Authors:** Zahra Hemati, Eleftherios Meletis, Abdollah Derakhshandeh, Masoud Haghkhah, Polychronis Kostoulas, Shoor Vir Singh, Kundan Kumar Chaubey, Saurabh Gupta

**Affiliations:** 1 Department of Pathobiology, School of Veterinary Medicine, Shiraz University, Shiraz, Iran; 2 Present address: Department of Pathobiology, School of Veterinary Medicine, ShahreKord University, ShahreKord, Iran; 3 Faculty of Public and One (Integrated) Health, School of Health Sciences, University of Thessaly, 43100 Karditsa, Greece; 4 Department of Biotechnology, Institute of Applied Sciences and Humanities, GLA University, Ajhai, Mathura, Uttar Pradesh 281406 India

**Keywords:** Paratuberculosis, IP-ELISA, Mt-ELISA, PCR-IS*900*/413, Culture, Test evaluation, Bayesian latent class models, Small ruminants

## Abstract

**Background:**

This study aimed to screen the sera of goats and sheep from flocks suspected of *Mycobacterium avium* subsp. *paratuberculosis* (MAP) infection by a newly standardized Mce-truncated ELISA (Mt-ELISA) kit for the detection of antibodies against MAP. Four diagnostic applied tests were evaluated including Indigenous plate-ELISA (IP-ELISA), Mt-ELISA, fecal Polymerase Chain Reaction (f-PCR) and fecal culture (FC).

**Materials and methods:**

Assuming the absence of a gold standard, latent-class models in a Bayesian framework were used to estimate the diagnostic accuracy of the four tests for MAP.

**Results:**

Mt-ELISA had higher Sensitivity (Se) in sheep (posterior median: 0.68 (95% Probability Interval (PI): 0.43–0.95), while IP-ELISA recorded the highest Se in goats as 0.83 (95% PI, 0.61–0.97). The f-PCR Se estimate slightly differed between species [sheep 0.36 (0.19–0.58), goats 0.19 (0.08–0.35)], while the Se of FC was similar between species [sheep 0.29 (0.15–0.51), goats 0.27 (0.13–0.45)]. The specificity estimates for all tests were high, close to unity, and similar between species.

**Conclusion:**

Overall, the results showed that the Mt-ELISA method can be used for MAP detection in small ruminants’ flocks.

**Supplementary Information:**

The online version contains supplementary material available at 10.1186/s12917-022-03141-7.

## Background

Paratuberculosis or Johne’s disease (JD) is chronic progressive enteritis primarily infecting ruminants, caused by an intracellular pathogen named *Mycobacterium avium* subsp. *paratuberculosis* (MAP) [1–3]. This Infection harms animal productivity (body weight, milk yield, feed conversion efficiency, fertility etc.) leading to huge economic losses to the dairy industry world-wide [4]. Unique MAP characteristics (extremely slow growth in artificial medium) and absence of adequate diagnostic tools due to variable serological response in early and late infection and variable shedding of MAP bacilli in feces in different age groups and physiological stages allow disease to thrive in small ruminants of the dairy industry [2, 5, 6]. Goats and sheep suffering from JD predominantly exhibit cachexia, reduced fertility, rough hair coat, skin roughness and soft feces without diarrhea [2, 3, 6, 7].

Infected animals are the cause of concern to public health since they are continuous sources of infection through their milk, colostrums and feces, recently classified as potential zoonotic [8–10]. Previous studies have shown some evidence of possible relationship between MAP infection and human disorders such as Crohn’s disease (CD) and Rheumatoid arthritis [10–13]. Also, an etiologic association between MAP infection and the development of CD has been reported in humans [14]. MAP infection in human tissue may display species-specific pathologic findings, as occurs with other zoonotic pathogens. Multiple studies reported live MAP bacilli isolated from milk and showed that the standard pasteurization method did not inactivate MAP bacilli, thereby classifying it as a food-borne pathogen [15–17]. Therefore, nowadays, motivation is directed towards developing new diagnostic and control methods to mitigate MAP infections in animals to reduce the risk of MAP transmission to humans.

Routinely, fecal and tissue culture, microscopy, ELISA based on purified protoplasmic antigen (PPA), and molecular tests (IS*900* PCR) are used for MAP infection detection [7, 18]. Fecal culture is considered the gold standard test for diagnosis in live animals [19]. However, the efficacy of this test is limited due to intermittent and low shedding of bacilli by infected animals and the long times needed for colonies to appear [5, 20]. The IS*900*- based assay is sensitive but depends on the stage of infection, as well as the quality and quantity of isolated DNA [21, 22]. For quick and economic screening, ELISA is the most popular test, though sensitivity (*Se*) and specificity (*Sp*) issues remain to be resolved [2, 23–26].

The prevalence of JD in goat herds and sheep flocks is unknown in many countries. The search for improved diagnostic tests and better control programs [6, 27] is a continuous process. In our previous study, we identified T and B cell antigenic regions on the C-terminal portion of the MAP2191 putative Mce protein (encoded by MAP2191 gene) using immuno-bioinformatics analysis and generated a recombinant truncated Mce protein for developing the Mce-truncated ELISA or Mt-ELISA method [28]. The Mce-family of proteins of pathogenic mycobacteria has been associated with virulence (enhanced invasiveness) and survival of MAP in macrophages and various cell lines of mammalian origin [16, 29]. Eight putative Mce-encoding genes were identified in the MAP strain’s K-10 genome [16, 29–31]. These proteins were detected by serum antibodies from MAP- infected animals supporting the hypothesis that the MAP Mce protein was immunogenic during infection [28].

Latent class models have been developed and applied in a series of situations to overcome the absence of a gold standard [32]. Furthermore, over the last decades, latent- class models have been implemented in a Bayesian framework [33, 34] that allows the incorporation of prior information. Due to the importance that live MAP bacilli represent for both humans and animals, a rapid, sensitive, and affordable diagnostic method that can ideally identify JD-infected animals is crucially needed. Removing JD-infected goats and sheep from flocks would eliminate the spread of this infection to other flocks and herds [21, 35]. Therefore, latent-class models in a Bayesian framework were used to estimate the diagnostic accuracy of the Mt-ELISA test. Specifically, the Mt-ELISA method was compared with fecal culture (FC), fecal PCR (f-PCR), and commercial IP-ELISA to maximize its applicability.

## Results

Fecal and serum samples 54 goats and 73 sheep were screened with four diagnostic tests mentioned above for MAP detection. The test results were dichotomized at the manufacturer’s suggested cut-off values and the cross-classified binary outcomes were used for the analysis. Tables 1 and 2 summarize the cross-classified results of IP-ELISA, Mt-ELISA, PCR-IS900/413, and culture. Typical MAP colonies were observed in eight (14.8%) out of 54 fecal samples of goats and 10 (13.7%) out of 73 fecal samples of sheep on HEY media slants after incubation at 37 °C. Of the 54 goat serum samples screened by Mt-ELISA and IP-ELISA, 25 (46.3%) and 28 (51.9%) were positive, respectively.

**Table 1:** Cross-classified results of IP-ELISA, Mt-ELISA, PCR-IS900/413 and culture (Sheep only)

				Mt- ELISA				Total
				Positive		Negative		–
				FC		FC		–
				Positive	Negative	Positive	Negative	–
	Positive	fPCR	Positive	6	1	0	0	7
**IP-ELISA**			Negative	1	9	0	10	20
	Negative	fPCR	Positive	0	2	1	3	6
			Negative	1	4	1	34	40
**Total**	–	–	–	8	16	2	47	73

PCR: Polymerase Chain Reaction.

FC: Fecal culture

**Table 2:** Cross-classified results of IP-ELISA, Mt-ELISA, PCR-IS900/413 and culture (Goats only)

				Mt- ELISA				Total
				Positive		Negative		–
				Culture		Culture		–
				Positive	Negative	Positive	Negative	–
	Positive	PCR	Positive	2	1	1	1	5
**IP-ELISA**			Negative	2	14	2	5	23
	Negative	PCR	Positive	0	0	0	2	2
			Negative	1	2	0	21	24
**Total**	–	–	–	5	17	3	29	54

Of the 73 sheep serum samples screened by Mt-ELISA and IP-ELISA, 24 (32.9%) and 27 (37%) were positive, respectively. The IS900-PCR showed that seven (13%) goat samples and 13 (17.8%) sheep samples were positive. Most of the sheep were more than 3 years old [1st quantile - 3, median – 5, 3rd quantile – 6], while the same holds for goats [1st quantile – 3, median – 4, 3rd quantile, 6].

Using multiple detection tests, 28 (22%) goats and sheep samples were positive for MAP infection cumulatively in the two antigen tests. Using the two antibody detection tests, 36 (28.4%) and 46 (36.2%) serum samples were positive in Mt-ELISA and IP-ELISA, respectively. However, two tests together detected 55 (43.3%) samples positive.

The posterior medians and 95% probability intervals (95% PIs) for all parameters of interest for both species are summarized in Table 3. The Mt-ELISA test had higher Se in sheep as 0.68 (95% PIs: 0.43–0.95), compared to IP-ELISA as 0.63 (0.42–0.83), f-PCR as 0.36 (0.19–0.58), and FC as 0.29 (0.15–0.51). On the other hand, IP-ELISA recorded the highest Se in goats as 0.83 (0.61–0.97), followed by Mt-ELISA as 0.63 (0.44–0.81), FC as 0.27 (0.13–0.45), and f-PCR as 0.19 (0.08–0.35). The reported Se of Mt-ELISA was similar between species [sheep 0.68 (0.43–0.95) and goats 0.63 (0.44–0.81)]. Further, the FC Se estimates were similar between species [sheep 0.29 (0.15–0.51) and goats 0.27 (0.13–0.45)]. Both IP-ELISA and f-PCR recorded slightly different Se estimates between species. The Se of IP-ELISA was higher in goats, while the Se of f-PCR was higher in sheep.

**Table 3:** Posterior medians and 95% probability intervals (PrIs) for the Sensitivity (Se) and the Specificity (Sp) of IP-ELISA, Mt-ELISA, f-PCR, Culture

Test		Sheep	Goats
	**Parameter**	**Posterior medians and 95%PrIs**	**Posterior medians and 95%PrIs**
**IP-ELISA**	Se	0.63 (0.42; 0.83)	0.83 (0.61; 0.97)
	Sp	0.92 (0.85; 0.97)	0.95 (0.9; 0.98)
**Mt-ELISA**	Se	0.68 (0.43; 0.95)	0.63 (0.44; 0.81)
	Sp	0.96 (0.91; 0.98)	0.95 (0.9; 0.98)
**f-PCR**	Se	0.36 (0.19; 0.58)	0.19 (0.08; 0.35)
	Sp	0.98 (0.94; 1)	0.98 (0.93; 1)
**Culture**	Se	0.29 (0.15; 0.51)	0.27 (0.13; 0.45)
	Sp	0.98 (0.95; 1)	0.99 (0.96; 1)
**IP-ELISA & Mt-ELISA**	cov-p^a^	0.04 (0; 0.12)	0.03 (0; 0.11)
	cov-n^b^	0.01 (0; 0.04)	0.02 (0; 0.05)
	cov-cdp^c^	0.19 (0.01; 0.55)	0.16 (0.01; 0.48)
	cov-cdn^d^	1.13 (0.05; 3.72)	1.81 (0.1; 4.31)
**f-PCR & Culture**	cov-p^a^	0.08 (0.02; 0.16)	0.04 (0; 0.11)
	cov-n^b^	0.004 (0; 0.02)	0.003 (0; 0.02)
	cov-cdp^c^	0.4 (0.09; 0.69)	0.24 (0.02; 0.55)
	cov-cdn^d^	2.21 (0.10; 7.22)	2.3 (0.1; 7.27)

^a^cov-p refers to the covariance term between sensitivities

^b^cov-n refers to the covariance term between specificities

^c^cov-cdp refers to covariance between sensitivities

^d^cov-cdn refers to covariance between specificities

The Sp estimates for all tests were high, close to unity, and similar between species. Specifically, the IP-ELISA Sp estimates were 0.92 (0.85–0.97) in sheep and 0.95 (0.9–0.98) in goats and, the Mt-ELISA Sp estimates were.96 (0.91–0.98) in sheep and, 0.95 (0.9–0.98) in goats. The f-PCR and FC tests recorded slightly higher Sp estimates [f-PCR: 0.98 (0.94–1) in sheep and, 0.98 (0.93–1) in goats; FC: 0.98 (0.95–1) in sheep and, 0.99 (0.96–1) in goats.

As indicated by the posterior medians and 95% PIs for the covariance terms conditional dependence existed between IP-ELISA and Mt-ELISA and between f-PCR and FC.

The results of uniform, uninformative prior distributions were unstable, even after restricting the parameter space; thus, they were not reported here. The results unrestricting the parameter space for the covariance terms are displayed in Supplementary Table 1 (ST1). The conditional dependence assumption between IP-ELISA and Mt-ELISA and between f-PCR and FC was examined unrestricting the parameter space to check if negative values were recorded. The Se and Sp estimates were comparable with ones acquired from the final model. However, covariance terms with negative 2.5th percentile values are reported. Specifically, the covariance term was adjusted for the conditional dependency between the Se estimates of IP-ELISA and Mt-ELISA. The model described in Section 2.8.3. was the one with the smallest DIC compared to the ones that were introduced in the Sensitivity Analysis section.

## Discussion

Johne’s disease (JD) inflicts huge economic losses to animal productivity and revealed the potential risk to human populations, as infected animals could shed huge quantities of causative agents in their feces, colostrum, and milk [17, 36]. Several ruminant species, especially sheep and goats, have been reported to be affected by MAP, and diagnosis of JD is extremely challenging due to the absence of a gold standard test [37]. The present study was set out for the first time to evaluate Mt-ELISA for the detection of MAP infection in selected Iranian small ruminant herds and flocks and also determined its accuracy by comparing with fecal culture, IP-ELISA, and molecular assays.

Studies are few on the diagnosis of JD prevailing in sheep and goat flocks and herds. Hendrick et al. (2005) reported moderate agreement between milk and serum ELISA, but milk ELISA results had a higher agreement with culture than serum ELISA [38]. Also, Angelidou et al. (2014) evaluated the milk ELISA via Bayesian validation and showed this ELISA was as accurate as the serum ELISA [39].

Pillai and Jayarao (2002) showed high Se of direct PCR with a detection limit of 10–100 CFU/ml pertaining to the use of large amounts of milk sample [40]. In our study, the rate of MAP detection was low in f-PCR, and the poor Se of f-PCR [0.36 (0.19–0.58)] was due to the low MAP bacillary count in fecal samples. The Actual performance of IS900-based PCR for the detection of MAP is limited by the quantity and quality of DNA and the presence of large quantities of inhibiting materials in clinical samples [41]. This result indicated that f-PCR cannot be reliably used on fecal samples with lower presence of MAP infection agents due to low Se. However, 10 culture-negative fecal samples were positive by f-PCR; therefore, these 10-FC negative samples may have contained MAP and were true positives but ruled out due to their inability to grow in HEY media. Based on the Sp of antigen detection tests, f-PCR [0.98 (0.93–1)] was highly specific.

Of the 127 animals screened, 18 (14.2%) were positive by FC and 49 (38.5%) were sero-positive with Mt-ELISA. Twelve out of 18 FC-positive animals (70%) could be detected by Mt-ELISA and only six out of 18 FC-positive animals (30.0%) could not be detected by Mt-ELISA. Also, Mt-ELISA has rated some of the animals as positive, whereas FC missed these animals for several reasons. These results may be attributed to the significant loss of viable MAP during HPC decontamination, the presence of low shedder animals or the disease status of each animal tested. Of the 49 sero-positives animals, only 12 (33.3%) were culture-positive. Lower detection in culture is due to lower Se of FC. Whereas due to higher Se, Mt and IP-ELISA showed though higher but true prevalence of MAP in the herds. Similarly Muskens et al. (2003), also showed that only low percentage of sero-positive animals (64 (17.3%) of 371) were confirmed as MAP shedders by FC [42]. In the study by Whitlock et al. (2000), about 25% of FC-negative cattle were considered infected because they had infected tissues at slaughter. Furthermore, it should be taken into account the difficulties MAP fecal culture in sheep and goats has in comparison with cattle [43].

In this study we used a Bayesian latent-class model adjusted for conditional dependence between IP-ELISA and Mt-ELISA and between f-PCR and FC. Further, within a Bayesian framework, the implementation of available prior information is possible. Even though in our setting identifiability conditions were held, informative prior distributions were introduced for the *Sps* of the applied methods to account for the sparsity of the observed data (i.e., zero cell observations – see Table 3), plus as indicated by the DIC that improved the fit of the model.

Overall, using both antigen and antibody combinations can distinguish between the true positive and false positive results in chronic infections like JD. Compared with culture results, the new Mt-ELISA method excluded apparently false-positive results of IP-ELISA. These differences were due to the fact that Mt-ELISA uses specific protein as antigen, where as in IP-ELISA, there is antigen mix of whole cell, therefore potentially detects animals in the different stages of incubating disease. These results highlighted potential capability of Mt-ELISA in preventing false-positive reactions that occur when a milieu of antigens is used in ELISA tests.

## Conclusion

Mt-ELISA identifies MAP specific antibodies in serum samples of infected small ruminants, while reducing the costs and time relative to those for standard mycobacterial culture, making Mt-ELISA practical test for screening of the suspected MAP infected animals in small ruminant herds. However, large scale studies still needed to be confirmed in real life and thus further studies are required.

## Methods

### Ethics approval and consent to participate

This study was approved by the Animal Ethics Committee (AECs) of School of Veterinary Medicine, Shiraz University (permit: 94GCU6M163973) and all the animal experiments were performed with our institutional guidelines and regulations (dated 20 September 2013) and ARRIVE guidelines for reporting animal research as much as possible (https://arriveguidelines.org/).

### Sheep and goats selection

Small ruminant herds included in our study were from states with endemic JD and a number of animals had clinical signs suggestive of MAP infections, and some animals had no suspicion of paratuberculosis. Ten small ruminant flocks were sampled randomly in late December 2018–March 2019. In this study, total numbers of animals to be analyzed were 54 goats and 73 sheep. At least 12 animals per herd were sampled. For each animal, age, gender and the flock of origin were noted based on information provided by the farmers. Fecal culture (FC), fecal PCR (f-PCR), Mce-truncated-ELISA (Mt-ELISA) and IP-ELISA was performed in all sampled animals.

### Feces and blood collection

Fecal samples (4–6 pellets) were collected by gloved hands directly from the rectum and blood samples (~ 5 ml) were collected from the jugular veins of each animal. Feces and blood samples were transported on ice directly to the bacteriology laboratory of the School of Veterinary Medicine of the Shiraz University, Iran. In the laboratory, fecal samples were processed freshly for MAP culture and f-PCR technique and blood samples are allowed to clot in tubes and sera were collected by centrifugation and were used for ELISA testing. All DNA samples and sera stored at − 70 °C prior to testing.

### Fecal culture

FC was performed using Herrold’s egg yolk (HEY) agar as per Singh et al., 2007a [19]. Approximately, 2 g of the feces were processed individually, first crushed inside the polyethylene sachets and then in sterile pestle and mortar with 5 ml sterilized 1X phosphate-buffered saline (1X PBS, pH 7.4). Then, fine paste of all samples was centrifuged (3500 rpm (rpm) for 45 min) and from three layers after centrifugation, supernatant layer is discarded, and semi-solid middle layer (over the top of the sediment) collected using a sterilized swab and subjected to decontamination using 20 ml of 0.9% hexadecylpyridinium chloride (HPC) for 18–24 h at room temperature. After incubation, the supernatant layer were carefully discarded, and a slant of HEY media was inoculated with 100 μL of remaining debris of each decontaminated fecal sample. A HEY medium slant inoculated with viable MAP and an uninoculated HEY media slant also used as controls to demonstrate media productivity and sterility respectively. All HEY slants were incubated at 37 °C and were screened weekly until the appearance of typical MAP colonies. A colony IS*900-*PCR was performed to confirm the MAP colonies.

### DNA isolation and f-PCR amplification

MAP genomic DNA from feces was extracted by chemical lysis followed by chloroform; Isoamyl alcohol purification. Purity and concentration of isolated DNA were determined Spectro-photometrically. The isolated DNA from each fecal sample was subjected to fecal-PCR (f-PCR) using MAP specific primers (P90/P91) targeting the IS*900* element, an insertion sequence unique to MAP as per Millar et al. (1996) [44]. Briefly, 5 μL of isolated fecal DNA was added to 20 μL of master mix (1 ul of P 90 & P 91 (10 pmol/μl), 1 unit of *Taq* Polymerase (5 U/*μ*l), MgCl_2_ (25.0 mM), dNTPs (2.0 mM), Buffer 10X). PCR reactions were performed (Bio-Rad, USA) and included an initial activation step at 95 °C for 10 min followed by 36 cycles of a 94 °C for 30 s, 58 °C for 20 s and 72 °C for 30 s. Fecal samples giving positive amplification for 413 bp product were considered as positive for MAP bacilli.

### Mce-truncated enzyme linked immunosorbent assay (Mt-ELISA)

Mt-ELISA was performed to quantify titer of the antibody in serum samples according to previously described by Hemati et al. [28]. Briefly, Flat bottom 96-well Microtiter were coated with 100 μl of recombinant Mce-truncated protein containing 0.02 μg of antigen in 0.5% carbonate-bicarbonate buffer (pH 9.6). Plates were then blocked with 100 μl / well 3.0% skimmed milk for 90 min at 37 °C. Following three washes with PBST, 100 μl of diluted serum (1:50) in 1.0% Bovine Serum Albumin (BSA) were added in duplicate to blocked plates and incubated at 37 °C for two hours. After washing (three times) with PBST, 100 μl of secondary anti-goat horseradish peroxidases conjugate (Cat. No. A8919, Sigma-Aldrich, USA) at 1:8000 dilution in 1X PBS was added to each well and incubated for 45 min at 37 °C. Finally, an absorbance of 450 nm was taken using an ELISA reader (Multiscan, Thermolab system, Finland).

### IP-ELISA

IP-ELISA kit, based on MAP semi-purified protoplasmic antigen (sPPA) prepared from the characterized strain (S 5) ‘Indian Bison Type’ bio-type of MAP of goat origin, was used as the manufacturer’s instructions (CIRG Lab, Makhdoom, Mathura, Uttar Pradesh, India) [45]. Briefly, Serum samples were used in 1:50 dilution and anti-species horseradish peroxidase conjugates (Sigma Aldrich, USA) at the dilution of 1:5000 for goats and sheep and 1:4000 for cattle and buffaloes in 1xPBS. Serum samples from culture positive and negative animals were used as positive and negative controls, respectively. Following incubation, an absorbance of 450 nm was taken using an ELISA reader (Multiscan, Thermolab system, Finland).

### Bayesian latent class analysis

In this study the STARD-BLCM reporting guidelines on the design, conduct and results of diagnostic accuracy studies that use BLCMs were adhered [46]. The STARD-BLCM checklist is available as a supplementary file.

#### Definition of infection status

In a Bayesian Latent Class analysis, the true infection status is considered latent i.e., hidden and depends on the condition/biomarker that the tests under evaluation target. The two ELISAs (Mt-ELISA, IP-ELISA) measure the host’s immune response (i.e., antibody presence or absence). On the other hand, both f-PCR and FC detect the infection agent itself or its products/debris. Therefore, “infection” means presence and persistence of MAP long enough to produce a detectable humoral immune response and sufficient shedding in feces. This definition of “infection” is similar with the one described in Nielsen et al. (2002) [47].

#### Model assumptions

In the absence of a gold standard, latent-class models [32] in a Bayesian framework were used to estimate the diagnostic accuracy of four tests for MAP: IP-ELISA, Mt-ELISA, f-PCR and FC. Given the Hui-Walter paradigm, in a two-tests two-populations setting, the main assumptions of a latent class model are that (a) the *Se* and the *Sp* of each test remain constant across all (sub) populations (b) all tests are conditionally independent of each other given the disease status and (c) a distinct difference between the (sub) populations’ true prevalence exists [48]). Our setting is a four-test one-population, thus, assumptions (a) and (c) are not applicable. Conditional independence between the two ELISAs cannot be assumed, on the basis that both methods measure the same biological process [49]. Therefore, covariance terms adjusting for conditional dependence were introduced, as described in Dendukuri and Joseph (2001) [50]. On the other hand, the two ELISAs can be considered conditional independent to f-PCR and FC [43]. Further, covariance terms adjusting for the conditional dependence between f-PCR and FC were introduced, even though studies assuming conditional independence between these two methods, are described in the literature [51]. Thus, all covariance terms introduced to the model were forced to be positive.

#### Model definition

Bayesian inference allows the estimation of the posterior distribution, which is the product of the prior information and the likelihood.

The model was constructed based on the assumption that the various test combinations follow the multinomial distribution. That is,

$$y\left[1:Q,1:Q,1:Q,1:Q\right]\sim dmulti\left(p\left[1:Q,1:Q,1:Q,1:Q\right],n\right)$$

where, *y* are the counts of various test combinations, *Q* = {1,2} the dichotomized test result (1 for positive, 2 for negative), *n* the population size and *p* the probability of observing each test combination, with the covariance terms introduced to adjust for the conditional dependence between IP-ELISA & Mt-ELISA and f-PCR & FC.

The number of parameters to be estimated are thirteen (13) for each species (i.e. four *Ses*, four *Sps,* four covariance terms and one pi), while the degrees of freedom offered by the data are fifteen (15). Therefore, identifiability criteria are met and a uniform, uninformative Beta prior distribution *Be* (1, 1) can be adopted for all parameters of interest. However, informative prior distributions for the Sps of the applied methods were used, to account for the sparsity of the observed data (i.e. zero cell observations – see Table 3). That is because, degrees of freedom being higher than the number of parameters to be estimated is a necessary, but not always sufficient condition to ensure identifiability. Sparse observed data diminish the ability of the model to estimate the associated parameters.

Prior Beta distributions were calculated for the *Sps* of the four tests using the PriorGen R package [52]. Specifically, f-PCR and FC were assumed to have a 0.98 mode (most probable value) with a 0.95 5th percentile, while the two ELISAs a 0.95 mode and 0.90 5th percentile. Detailed justification on the selection of the prior distributions is provided in Kostoulas et al. (2006) [32].

The same model was run for both species; sheep and goats. Parameter estimates were based on analytical summaries of 100,000 iterations of two chains, after a burn-in phase of 5000 iterations. All checks suggested that convergence occurred and autocorrelations dropped-off fast. Models were run in the freeware program OpenBUGS [53].

#### Sensitivity analysis

The model was run with uniform, uninformative Be (1,1) prior distributions, to assess the influence of prior information. Further, to validate the conditional dependence assumption between IP-ELISA & Mt-ELISA and f-PCR & FC the parameter space was unrestricted for the covariance terms, to monitor if negative values are recorded. Model selection was based on the DIC (Deviance Information Criterion) dialog box in OpenBUGS environment. The model with the smallest DIC is the model that best fits the data i.e., the model that would best predict a replicate dataset of the same structure as the currently observed [54].

## Supplementary Information

**Additional file 1: Supplementary Table 1.** Posterior medians and 95% probability intervals (PrIs) for the Sensitivity (Se) and the Specificity (Sp) of IP-ELISA, Mt-ELISA, f-PCR, Culture.

## Data Availability

The data sets used and/or analyzed during the current study are available from the corresponding author on reasonable request.

## References

[1] Plain KM, Marsh IB, Waldron AM, Galea F, Whittington AM, Saunders VF, Begg DJ, de Silva K, Purdie AC, Whittington RJ (2014). High-throughput direct fecal PCR assay for detection of *Mycobacterium avium* subsp. *paratuberculosis* in sheep and cattle. J Clin Microbiol.

[2] Koets A, Ravesloot L, Ruuls R, Dinkla A, Eisenberg S, Lievaart-Peterson K (2019). Effects of age and environment on adaptive immune responses to *Mycobacterium avium* subsp. *paratuberculosis* (MAP) vaccination in dairy goats in relation to paratuberculosis control strategies. Vet Sci.

[3] Grossi DL, Santori D, Barone A, Abbruzzese S, Ricchi M, Marcario AG (2020). Isolation of *Mycobacterium avium* subsp. *Paratuberculosis* in the Feces and Tissue of Small Ruminants Using a Non-Automated Liquid Culture Method. Animals (Basel).

[4] Rawat KD, Chaudhary S, Kumar N, Gupta S, Chaubey KK, Singh SV, Dhama K, Verma AK, Deb R (2014). Economic losses in a commercial dairy farm due to the out-break of Johne’s disease in India. Res J Vet Pract.

[5] Mathevon Y, Foucras G, Falguières R, Corbiere F (2017). Estimation of the sensitivity and specificity of two serum ELISAs and one fecal qPCR for diagnosis of paratuberculosis in sub-clinically infected young adult French sheep using latent class Bayesian modeling. BMC Vet Res.

[6] Barrero-Domínguez B, Luque I, Huerta B, Gomez-Laguna J, Galán-Relaño Á, Gómez-Gascón L, et al. Paratuberculosis in dairy goat flocks from southern Spain: risk factors associated with seroprevalence. Vet Rec. 2019;185(19). 10.1136/vr.105465 31530721

[7] Arsenault J, Singh Sohal J, Leboeuf A, Hélie P, Fecteau G, Robinson Y (2019). L’Homme Y. validation of an in-house real-time PCR fecal assay and comparison with two commercial assays for the antemortem detection of *Mycobacterium avium* subsp. *paratuberculosis* infection in culled sheep. J Vet Diagn Investig.

[8] Kuenstner L, Kuenstner JT (2021). *Mycobacterium avium* ssp. *paratuberculosis* in the food supply: a public health issue. Front. Public Health.

[9] Chaubey KK, Singh SV, Gupta S, Singh M, Sohal JS, Kumar N, Singh MK, Bhatia AK, Dhama K (2017). *Mycobacterium avium* subsp. *paratuberculosis* - an important food borne pathogen of high public health significance with special reference to India: an update. Vet Quart.

[10] Lowe AM, Yansouni CP, Behr MA (2008). Causality and gastrointestinal infections: Koch, hill, and Crohn's. Lancet Infect Dis.

[11] McNees AL, Markesich D, Zayyani NR, Graham DY (2015). *Mycobacterium paratuberculosis* as a cause of Crohn's disease. Expert Rev Gastroenterol Hepatol.

[12] Singh S.V, Kuenstner JT, Davis WC, Agarwal P, Kumar N, Singh D, Gupta S, Chaubey KK, Tyagi AK, Misri J, Jayaraman S, Sohal JS and Dhama K. Concurrent resolution of chronic diarrhea likely due to Crohn's disease and infection with *Mycobacterium avium paratuberculosis*. Front Med 2016;3:49. 10.3389/fmed.2016.00049 PMC5081375 27833911

[13] Bo M, Erre GL, Bach H, Slavin YN, Manchia PA, Passiu G, Sechi LA (2019). PtpA and PknG Proteins Secreted by *Mycobacterium avium* subsp. *paratuberculosis* are Recognized by Sera from Patients with Rheumatoid Arthritis: A Case–Control Study. J Inflam Res.

[14] Zarei-Kordshouli F, Geramizadeh B, Khodakaram-Tafti A (2019). Prevalence of *Mycobacterium avium* subspecies *paratuberculosis* IS 900 DNA in biopsy tissues from patients with Crohn's disease: histopathological and molecular comparison with Johne's disease in Fars province of Iran. BMC Infect Dis.

[15] Dzieciol M, Volgger P, Khol J, Baumgartner W, Wagner M, Hein I (2010). A novel real-time PCR assay for specific detection and quantification of *Mycobacterium avium* subsp. *paratuberculosis* in milk with the inherent possibility of differentiation between viable and dead cells. BMC Res Notes.

[16] Hemati Z, Haghkhah M, Derakhshandeh A, Singh S, Chaubey KK (2018). Cloning and characterization of MAP2191 gene, a mammalian cell entry antigen of Mycobacterium avium subspecies paratuberculosis. Mol Bio Res Commun.

[17] Machado D, Lecorche E, Mougari F, Cambau E, Viveiros M (2018). Insights on mycobacterium leprae efflux pumps and their implications in drug resistance and virulence. Front Microbiol.

[18] O’Brien DP, Robson M, Friedman ND, Walton A, McDonald A, Callan P, Hughes A, Rahdon R, Athan E (2013). Incidence, clinical spectrum, diagnostic features, treatment and predictors of paradoxical reactions during antibiotic treatment of *mycobacterium ulcerans* infections. BMC Infect Dis.

[19] Singh SV, Singh AV, Singh R, Sandhu KS, Singh PK, Sohal JS, Gupta VK, Vihan VS (2007). Evaluation of highly sensitive indigenous milk ELISA kit with fecal culture, milk culture and fecal-PCR for the diagnosis of bovine Johne’s disease (BJD) in India. Comp Immunol Microbiol Infect Dis.

[20] Schwalm AK, Obiegala A, Pfeffer M, Sting R (2018). Enhanced sensitivity and fast turnaround time in laboratory diagnosis for bovine paratuberculosis in fecal samples. J Microbiol Methods.

[21] Prendergast BJ, Onishi KG, Zucker I (2014). Female mice liberated for inclusion in neuroscience and biomedical research. Neurosci Biobehav Rev.

[22] Keller SM, Stephan R, Kuenzler R, Meylan M, Wittenbrink MM (2014). Comparison of fecal culture and F57 real-time polymerase chain reaction for the detection of *Mycobacterium avium* subspecies *paratuberculosis* in Swiss cattle herds with a history of paratuberculosis. Acta Vet Scand.

[23] Whitlock RH, Wells SJ, Sweeney RW, Van Tiem J (2000). ELISA and fecal culture for paratuberculosis (Johne’s disease): sensitivity and specificity of each method. Vet Microbiol.

[24] McKenna SL, Keefe GP, Barkema HW, Sockett DC (2005). Evaluation of three ELISAs for Mycobacterium avium subsp. paratuberculosis using tissue and fecal culture as comparison standards. Vet Microbiol.

[25] Clark S, Cross ML, Nadian A, Vipond J, Court P, Williams A, Hewinson RG, Aldwell FE, Chambers MA (2008). Oral vaccination of guinea pigs with a *Mycobacterium bovis* bacillus Calmette-Guerin vaccine in a lipid matrix protects against aerosol infection with virulent *M. bovis*. Infect Immun.

[26] Costanzo G, Pinedo FA, Mon ML, Viale M, Gil A, Illia MC, et al. Accuracy assessment and screening of a dairy herd with paratuberculosis by three different ELISAs. Vet Microbiol. 2012;23;156(1-2):183-8. 10.1016/j.vetmic.2011.10.029 22138619

[27] Munjal SK, Tripathi BN, Paliwal OP (2005). Progressive immunopathological changes during early stages of experimental infection of goats with Mycobacterium avium subspecies paratuberculosis. Vet Pathol.

[28] Hemati Z, Haghkhah M, Derakhshandeh A, Chaubey KK, Singh SV (2020). Novel recombinant Mce-truncated protein based ELISA for the diagnosis of *Mycobacterium avium* subsp. *paratuberculosis* infection in domestic livestock. PLoS One.

[29] Hemati Z, Derakhshandeh A, Haghkhah M, Chaubey KK, Gupta S, Singh M, Singh SV, Dhama K (2019). Mammalian cell entry operons; novel and major subset candidates for diagnostics with special reference to *Mycobacterium avium* subspecies *paratuberculosis* infection. Vet Quarterl.

[30] Paustian ML, Zhu X, Sreevatsan S, Robbe-Austerman S, Kapur V, Bannantine JP (2008). Comparative genomic analysis of Mycobacterium avium subspecies obtained from multiple host species. BMC Genomics.

[31] Castellanos E, Aranaz A, De Juan L, Álvarez J, Rodríguez S, Romero B, Bezos J, Stevenson K, Mateos A, Domínguez L (2009). Single nucleotide polymorphisms in the IS900 sequence of Mycobacterium avium subspecies paratuberculosis are strain type specific. J Clin Microbiol.

[32] Hui SL, Walter SD (1980). Estimating the error rates of diagnostic tests. Biometrics..

[33] Enøe C, Andersen S, Sørensen V, Willeberg P (2001). Estimation of sensitivity, specificity and predictive values of two serologic tests for the detection of antibodies against *Actinobacillus pleuropneumoniae* serotype 2 in the absence of a reference test (gold standard). Prev Vet Med..

[34] Branscum AJ, Gardner IA, Johnson WO (2005). Estimation of diagnostic-test sensitivity and specificity through Bayesian modeling. Prev Vet Med.

[35] Küntzel A, Weber M, Gierschner P (2019). Core profile of volatile organic compounds related to growth of Mycobacterium avium subspecies paratuberculosis - a comparative extract of three independent studies. PLoS One.

[36] Bates, A., O’Brien, R., Liggett, S. et al. Control of Mycobacterium avium subsp. paratuberculosis infection on a New Zealand pastoral dairy farm. BMC Vet Res 15, 266 (2019). https://doi.org/10.1186/s12917-019-2014-6 10.1186/s12917-019-2014-6 PMC6664707 31358004

[37] Buczinski S, Arsenault J, Kostoulas P, Corbière F, Fecteau G, Dendukuri N (2019). Accuracy of paratuberculosis diagnostic tests in small ruminants: protocol for a systematic review and meta-analysis. Anim Health Res Rev.

[38] Hendrick S, Duffield T, Leslie K, Lissemore K, Archambault M, Kelton D (2005). The prevalence of milk and serum antibodies to *Mycobacterium avium* subspecies *paratuberculosis* in dairy herds in Ontario. Can Vet J.

[39] Angelidou E, Kostoulas P, Leontides L (2014). Bayesian validation of a serum and milk ELISA for antibodies against *Mycobacterium avium* subspecies *paratuberculosis* in Greek dairy goats across lactation. J Dairy Sci.

[40] Pillai SR, Jayarao BM (2002). Application of IS900 PCR for detection of *Mycobacterium avium* subsp. *paratuberculosis* directly from raw milk. J Dairy Sci.

[41] Khare S, Ficht TA, Santos RL, Romano J, Ficht AR, Zhang S, Grant IR, Libal M, Hunter D, Adams LG (2004). Rapid and sensitive detection of Mycobacterium avium subsp. paratuberculosis in bovine milk and feces by a combination of immunomagnetic bead separation-conventional PCR and real-time PCR. J Clin Microbiol.

[42] Muskens J, Mars MH, Elbers ARW, Van Maanen K, Bakker D (2003). The results of using fecal culture as confirmation test of paratuberculosis-seropositive dairy cattle. J vet med. B infect dis vet. Public Health.

[43] Kostoulas P, Leontides L, Enøe C, Billinis C, Florou M, Sofia M (2006). Bayesian estimation of sensitivity and specificity of serum ELISA and faecal culture for diagnosis of paratuberculosis in Greek dairy sheep and goats. Prev Vet Med..

[44] Millar D, Ford J, Sanderson J, Withey S, Tizard M, Doran T, Hermon-Taylor J (1996). IS900 PCR to detect *mycobacterium paratuberculosis* in retail supplies of whole pasteurized cows’ milk in England and Wales. Appl Environ Microbiol.

[45] Singh SV, Singh AV, Singh PK, Sohal JS, Singh NP (2007). Evaluation of an indigenous ELISA for diagnosis of Johne’s disease and its comparison with commercial kits. Indian J Microbiol.

[46] Kostoulas P, Nielsen SS, Branscum AJ, Johnson WO, Dendukuri N, Dhand NK, Toft N, Gardner IA (2017). STARD-BLCM: standards for the reporting of diagnostic accuracy studies that use Bayesian latent class models. Prev Vet Med..

[47] Nielsen SS, Grønbæk C, Agger JF, Houe H (2002). Maximum-likelihood estimation of sensitivity and specificity of ELISAs and faecal culture for diagnosis of paratuberculosis. Prev Vet Med..

[48] Toft N, Jørgensen E, Højsgaard S (2005). Diagnosing diagnostic tests: evaluating the assumptions underlying the estimation of sensitivity and specificity in the absence of a gold standard. Prev Vet Med..

[49] Gardner IA, Stryhn H, Lind P, Collins MT (2000). Conditional dependence between tests affects the diagnosis and surveillance of animal diseases. Prev Vet Med..

[50] Dendukuri N, Joseph L (2001). Bayesian approaches to modeling the conditional dependence between multiple diagnostic tests. Biometrics..

[51] Alinovi CA, Ward MP, Lin TL, Moore GE. Wu CC. Real-time PCR, compared to liquid and solid culture media and ELISA, for the detection of *Mycobacterium avium* ssp. *paratuberculosis*. Vet Microbiol 2009;136(1–2): 177–179. 10.1016/j.vetmic.2008.10.012 19091493

[52] Kostoulas P . PriorGen: Generates Prior Distributions for Proportions. R package version. 2018; 1(2).

[53] Spiegelhalter D, Thomas A, Best N, Lunn D (2003). WinBUGS User Manual.

[54] Spiegelhalter D, Best N, Carlin P, Van Der Linde A (2002). Bayesian measures of model complexity and fit. J R Stat Soc Series b (Stat Methodol).

