# Supplementary material for: Application of Bayesian modeling for diagnostic assays of Mycobacterium avium subsp. paratuberculosis in sheep and goats flocks
Source: BMC Vet Res. 2022 Jan 18;18:47. doi: 10.1186/s12917-022-03141-7 (PMC8764775; doi:10.1186/s12917-022-03141-7)
Supplement: Supplementary file 1 — Additional file 1: Supplementary Table 1. Posterior medians and 95% probability intervals (PrIs) for the Sensitivity (Se) and the Specificity (Sp) of IP-ELISA, Mt-ELISA, f-PCR, Culture. [file 12917_2022_3141_MOESM1_ESM.docx]

**Supplementary Table 1.** Posterior medians and 95% probability intervals (PrIs) for the Sensitivity (Se) and the Specificity (Sp) of IP-ELISA, Mt-ELISA, f-PCR, Culture.

| **Test** |  | **Sheep** | **Goats** |
| --- | --- | --- | --- |
|  | **Parameter** | **Posterior medians and 95%PrIs** | **Posterior medians and 95%PrIs** |
| **IP-ELISA** | Se | 0.66 (0.45; 0.88) | 0.86 (0.65; 0.97) |
|  | Sp | 0.93 (0.86; 0.98) | 0.95 (0.9; 0.98) |
| **Mt-ELISA** | Se | 0.69 (0.45; 0.95) | 0.66 (0.46; 0.82) |
|  | Sp | 0.95 (0.86; 0.98) | 0.95 (0.9; 0.98) |
| **f-PCR** | Se | 0.34 (0.18; 0.56) | 0.19 (0.08; 0.35) |
|  | Sp | 0.97 (0.93; 1) | 0.97 (0.93; 1) |
| **Culture** | Se | 0.29 (0.14; 0.5) | 0.27 (0.14; 0.44) |
|  | Sp | 0.98 (0.95; 1) | 0.99 (0.96; 1) |
| **IP-ELISA & Mt-ELISA** | cov-p^a^ | 0.02 (-0.05; 0.11) | 0.01 (-0.04; 0.01) |
|  | cov-n^b^ | 0.01 (0; 0.04) | 0.02 (0; 0.05) |
|  | cov-cdp^c^ | 0.11 (-0.25; 0.53) | 0.05 (-0.24; 0.45) |
|  | cov-cdn^d^ | 0.97 (-0.26; 3.77) | 1.78 (-0.17; 4.33) |
| **f-PCR & Culture** | cov-p^a^ | 0.08 (0.02; 0.16) | 0.04 (-0.01; 0.11) |
|  | cov-n^b^ | 0.004 (0; 0.02) | 0.003 (0; 0.02) |
|  | cov-cdp^c^ | 0.43 (0.08; 0.73) | 0.25 (-0.07; 0.58) |
|  | cov-cdn^d^ | 1.88 (-0.07; 6.72) | 2.09 (-0.07; 6.82) |

^a^cov-p refers to the covariance term between sensitivities

^b^cov-n refers to the covariance term between specificities

^c^covcdp refers to covariance between sensitivities

^d^covcdn refers to covariance between specificities

|  | **Section & Topic** | **No** | **Item** | **Reported on page #** |
| --- | --- | --- | --- | --- |
|  |  |  |  |  |
|  | **TITLE OR ABSTRACT** |  |  |  |
|  |  | **1** | Identification as a study of diagnostic accuracy, using at least one measure of accuracy (such as sensitivity, specificity, predictive values, or AUC) **and Bayesian latent class models** | **1** |
|  | **ABSTRACT** |  |  |  |
|  |  | **2** | Structured summary of study design, methods, results, and conclusions  (for specific guidance, see STARD for Abstracts) | **1** |
|  | **INTRODUCTION** |  |  |  |
|  |  | **3** | Scientific and clinical background, including the intended use and clinical role of the **tests under evaluation** | **2 - 4** |
|  |  | **4** | Study objectives and hypotheses, **such as estimation of diagnostic accuracy of the tests for a defined purpose through BLCM** | **4** |
|  | **METHODS** |  |  |  |
|  | *Study design* | **5** | Whether data collection was planned before the **tests** were performed (prospective study) or after (retrospective study) | **5** |
|  | *Participants* | **6** | Eligibility criteria **and description of the source population** | **5** |
|  |  | **7** | On what basis potentially eligible participants were identified  (such as symptoms, results from previous tests, inclusion in registry) | **5** |
|  |  | **8** | Where and when potentially eligible participants were identified (setting, location and dates) | **5** |
|  |  | **9** | Whether participants formed a consecutive, random or convenience series | **5** |
|  | *Test methods* | **10** | **Description of the tests under evaluation**, in sufficient detail to allow replication, **and/or cite references** | **5 – 8** |
|  |  | **11** | Rationale for choosing the **tests under evaluation in relation to their purpose** | **5 – 8** |
|  |  | **12** | Definition of and rationale for test positivity cut-offs or result categories of **the tests under evaluation**, distinguishing pre-specified from exploratory | **8** |
|  |  | **13** | Whether clinical information was available to the performers or readers of **the tests under evaluation** | **5 – 8** |
|  | *Analysis* | **14a** | **BLCM model** for estimating measures of diagnostic accuracy | **8 – 12** |
|  |  | **14b** | **Definition and rationale of prior information and sensitivity analysis** | **11** |
|  |  | **15** | How indeterminate results **of the tests under evaluation** were handled | **No indeterminate results** |
|  |  | **16** | How missing data **of the tests under evaluation** were handled | **No missing data** |
|  |  | **17** | Any analyses of variability in diagnostic accuracy, distinguishing pre-specified from exploratory | **8 - 12** |
|  |  | **18** | Intended sample size and how it was determined | **5** |
|  | **RESULTS** |  |  |  |
|  | *Participants* | **19** | Flow of participants, using a diagram | **NA** |
|  |  | **20** | Baseline demographic and clinical characteristics of participants | **NA** |
|  |  | **21** | **The distribution of the targeted conditions is unknown, hence the use of BLCM** | **Not applicable** |
|  |  | **22** | Time interval and any clinical interventions between **the tests under evaluation** | **Not applicable** |
|  | *Test results* | **23** | Cross tabulation of the **tests’ results (or for continuous tests results their distribution by infection stage)** | **Table 3** |
|  |  | **24** | Estimates of diagnostic accuracy **under alternative prior specification** and their precision (such as 95% **credible/probability intervals**) | **Tables 5 & Supplementary Table 1** |
|  |  | **25** | Any adverse events from performing **the tests under evaluation** | **Not applicable** |
|  | **DISCUSSION** |  |  |  |
|  |  | **26** | Study limitations, including sources of potential bias, statistical uncertainty, and generalisability | **13 – 15** |
|  |  | **27** | Implications for practice, including the intended use and clinical role of **the tests under evaluation in relevant settings (clinical, research, surveillance etc.)** | **13 – 15** |
|  | **OTHER INFORMATION** |  |  |  |
|  |  | **28** | Registration number and name of registry | **Not applicable** |
|  |  | **29** | Where the full study protocol can be accessed | **Not applicable** |
|  |  | **30** | Sources of funding and other support; role of funders | **16** |
|  |  |  |  |  |

STARD - BLCM

STARD-BLCM stands for “Standards for the Reporting of Diagnostic accuracy studies that use Bayesian Latent Class Models” and is a modification of the STARD statement (which was recently updated to STARD2015). STARD-BLCM aims to facilitate improved quality of reporting for diagnostic accuracy studies that use Bayesian latent class models in the absence of a reference standard. The proposed modifications are relevant to both Bayesian and frequentist estimation methods but the focus is on the former.

More information for STARD (STARD2015) can be found at: <http://www.equator-network.org/reporting-guidelines/stard>

More information for STARD-BLCM can be found at: <http://www.equator-network.org/reporting-guidelines/stard-blcm>
